# Airflow obstruction as a marker of adverse prognosis in rheumatoid arthritis

**DOI:** 10.3389/fmed.2023.1063012

**Published:** 2023-03-09

**Authors:** Julien Guiot, Monique Henket, Marie Ernst, Laurence Seidel, Marie Winandy, Anna Denis, Anne-Noëlle Frix, Fanny Gester, Marie Thys, Laurie Giltay, Omaima Garah, Makon-Sébastien Njock, Perrine Canivet, Paul Meunier, Jean-Louis Corhay, Céline Regnier, Olivier Malaise, Michel Malaise, Renaud Louis

**Affiliations:** ^1^Department of Respiratory Medicine, CHU Liège, Liège, Belgium; ^2^Biostatistics and Research Method Center (B-STAT), CHU Liège, Liège, Belgium; ^3^Department of Medico-Economic and Data, CHU Liège, Liège, Belgium; ^4^Department of Radiology, CHU Liège, Liège, Belgium; ^5^Department of Rheumatology, CHU Liège, Liège, Belgium

**Keywords:** rheumatoid arthritis, airway obstruction, chronic obstructive pulmonary disease, interstitial lung diseases, bronchiolitis

## Abstract

**Objectives:**

In our study, we explored the specific subgroup of patients with rheumatoid arthritis (RA) suffering from obstructive lung disease (OLD) and its impact on morbi-mortality.

**Methods:**

Our retrospective study included 309 patients suffering from RA with either obstructive (O-RA) or non-obstructive patterns (non-O-RA). OLD was defined based on the Tiffeneau index at the first available pulmonary functional test (PFT). Survival was then calculated and represented by a Kaplan–Meier curve. The comparison between the populations considered was performed by the Log-Rank test.

**Results:**

Out of the 309 RA patients, 102 (33%) had airway obstruction. The overall survival time was significantly lower in the O-RA group than in the non-O-RA group (*n* = 207) (*p* < 0.001). The median survival time was 11.75 years in the O-RA group and higher than 16 years in the non-O-RA group. Multivariate analysis identified OLD as an independent risk factor for mortality (HR 2.20; 95% CI 1.21–4.00, *p* < 0.01).

**Conclusion:**

Airway obstruction can be an independent risk factor of mortality in RA and should be considered as an early marker of poor prognosis. Further prospective longitudinal studies are required in order to determine the best clinical management for O-RA patients.

## Introduction

Rheumatoid arthritis is a systemic inflammatory disorder that most commonly affects the joints, causing progressive destruction of the cartilage and the bone, which reduces mobility and can lead to severe functional disabilities. Apart from the musculoskeletal disease, one of the main manifestations driving its mortality is interstitial lung disease (ILD) widely known to be associated with a worse outcome in this context (1).

Patients suffering from RA typically have circulating antibodies, the most common being rheumatoid factor (RF) and anti-cyclic citrullinated peptide (CCP). Antibodies onset may precede the disease onset and may be associated with the development of RA-associated lung disease. Physiopathologically, there is growing evidence that RA begins in the lung with active citrullination of vimentin peptides induced by tobacco exposure (2, 3). Similarly, epidemiological data showed that in a global RA population, most of the patients were presenting a tobacco abuse history (former smokers or ex-smokers) (4).

Out of this context, chronic smoking abuse is widely recognized to induce chronic obstructive pulmonary disease (COPD) and emphysema. COPD is an obstructive lung disease (OLD) where airflow limitation is not fully reversible (5). Functional definition is based on spirometry and a post-bronchodilator FEV1/FVC ratio (Tiffeneau index) of less than 70%, which is the usually recommended cut-off (5). The origin of airflow obstruction is thought to be related to persistent bronchial inflammation due to noxious particles and gas.

The most frequently incriminated etiologic factor is tobacco smoking and environmental exposure. In opposition, RA is frequently associated with ILD and is clinically significant in up to 10% of the global RA population which leads to a restrictive syndrome defined as a reduced total lung capacity (TLC) (6–10). Some patients can experience an association of those two conditions that can modify the overall morbi-mortality of those patients (1, 6–8, 10). According to different studies, the percentages of RA patients with COPD vary from 3 to 10% (10), patients with bronchiectasis, from 2 to 58% (11, 12), and patients with ILD (either nonspecific interstitial pneumonia (NSIP) or usual interstitial pneumonia (UIP)) from 6 to 56% (9, 12).

Therefore, the evaluation of OLD associated with RA is a key question to determine its impact on patient mortality. It is important to note that patients suffering from RA can underestimate respiratory symptoms due to a significant reduction in their mobility induced by arthritis and inflammatory symptoms.

This descriptive and explorative study aimed to describe RA patients exhibiting OLD (O-RA) phenotype and their functional characteristics compared to those without obstruction.

## Methods

### Study population

A retrospective observational study was conducted. Patients were recruited from our ambulatory care policlinic at CHU from 01-08-2004 to 01-01-2020 based on a systematic evaluation of electronic hospital records using specific keywords (rheumatoid arthritis). We selected patients with available PFT suffering from RA according to ACR/EULAR 2010 classification criteria for RA (13) based on a specialized evaluation by a rheumatologist. We did not exclude patients based on clinical criteria or comorbidities.

Airway obstruction was defined by a Tiffeneau index (FEV1/FVC assessed after salbutamol 400 μg) < 70% (5). Progressive fibrosing ILD (PF-ILD) was defined as:

- a relative decline in FVC of at least 10% of the predicted value;
- or a relative decline in the FVC of 5% to less than 10% of the predicted value and worsening of respiratory symptoms;
- or an increased extent of fibrosis on high-resolution computed tomography (HRCT) of the chest;
- or worsening of respiratory symptoms and an increased extent of fibrosis on HRCT.

Asthma was evaluated as recommended by GINA guidelines (14), whereas specific documentation was manually obtained from the patient’s medical file.

### Collected data

Information was collected on patients’ characteristics (age, gender, smoking status) and clinical characteristics (diagnosis, medical history, radiological patterns, lung function, biomarkers, and treatment). We were not able to collect cardiovascular comorbidities, known to be of interest in the RA population, nor common comorbidities such as diabetes, renal or hepatic failure, and arterial hypertension, from the patient’s medical files, because this study was retrospective, and we did not have a reliable and dedicated evaluation of these parameters.

Data on medications were collected throughout the study, including immunosuppressive agents. The survival status of the patients was confirmed based on a specific analysis of the national registry.

PFT values were the earliest available and the biological values [hemoglobin, white blood cell count, platelets, C-reactive protein (CRP), fibrinogen, lymphocytes, monocytes, neutrophils, anti-citrullinated peptide antibody (ACPA), and rheumatoid factor (RF)] were the values closest to the date of the first PFT (±1 month).

The protocol was approved by the ethics committee of the University Hospital of Liège (Belgian Number: B707201422832; ref.: 2022/52).

### Patient and public involvement

Patients or the public were not involved in our research’s design, conduct, reporting, or dissemination plans.

### Statistics

Results are presented as frequency tables for qualitative variables and as mean and standard deviation (SD) or as the median and interquartile range (IQR) for quantitative variables.

Comparisons between O-RA and non-O-RA groups were done by chi-square tests (or Fisher exact) for categorical variables, or by Student *t*-test for continuous variables (log-transformed data in case of skewness). Overall survival since the first available PFT was represented by a Kaplan–Meier curve. Cox regression models were used to analyze the overall survival concerning groups and other parameters (FVC, DLCO, FEV-1, Age, gender, RA-ILD, bronchiectasis, emphysema, asthma, neoplasia, and COPD). Results were considered significant at the 5% uncertainty level (*p* < 0.05). The calculations were performed using SAS version 9.4 and the graphs using R version 4.1.0.

## Results

### Subject demographics

Among our cohort of 1,497 retrospectively collected patients suffering from RA, 309 had at least one PFT available; 299 out of these 309 patients also had an HRCT. In this cohort, 102 patients were exhibiting the obstructive phenotype (33%). Patients were predominantly female, with 54 and 72% for O-RA and non-O-RA, respectively. The mean age was of 67 ± 10 years for the O-RA group. 69% of the non-O-RA and 76% of the O-RA group were active or former smokers. There was no significant difference between the two groups regarding smoking status.

Among patients treated with immunosuppressors, 70% were non-O-RA. Patients who suffered from O-RA were less likely to be affected by ILD. The obstructive phenotype of their disease was more often manifested by the presence of asthma, COPD, emphysema, or neoplasia. Patients with O-RA benefited more frequently from bronchodilator therapy and inhaled corticotherapy (Table 1).

**Table 1:** Patient characteristics.

**Characteristic**	**Non-O-RA *n* = 207**	**O-RA *n* = 102**
Demography		
Age, years	62 ± 12	67 ± 10^**^
Gender (M/F)	58/149	47/55^**^
BMI, Kg/m^2^	26 ± 5	25 ± 6
Smokers NS/FS/CS (%)	31 - 51 - 18	24 - 59 - 17
Age at the first visit to rheumatology, years ^a^	57 ± 13	62 ± 10^**^
Duration of RA, years ^a^	5.12 ± 4.63	4.43 ± 4.2
Death, yes (%)	29 (14%)	36 (35%)
RA-associated lung abnormalities		
Lung abnormalities	99 (48%)	44 (43%)
ILD^b^	59 (28%)	18 (18%)^*^
PF-ILD	38 (64%)	7 (39%)^**^
NSIP	36 (61%)	10 (56%)
UIP	19 (32%)	4 (22%)
COP	2 (3%)	0 (0%)
Nodule	17 (8%)	14 (14%)
Bronchiectasis	56 (27%)	21 (20%)
With ILD	26 (46%)	6 (33%)
Emphysema	2 (1%)	26 (26%)^****^
Asthma ^c^	14 (7%)	16 (16%)^**^
COPD ^d^	0	76 (78%)^****^
ACOS	0	7 (7%)
Obstructive syndrome	0	16 (16%)^****^
Pulmonary neoplasia at T0 ^e^	2 (1%)	5 (5%)^*^
Erosive RA ^f^	74 (73%)	28 (65%)
Treatments		
Immunosuppressor (DMARD) yes/no (%)	115/73 (61%)	49/42 (54%)
Biologic IS		
Anti TNF, yes(%)	10 (9%)	9 (18%)
Other, yes(%)	18 (16%)	3 (6%)
Synthetic IS yes (% IS)	55 (48%)	30 (61%)
Immunomodulator, yes(%)	55 (48%)	30 (61%)
Combined IS with immunomodulator		
With anti-TNF, yes(%)	22 (19%)	7 (14%)
Other, yes(%)	10 (9%)*	0
OCS	75 (36%)	55 (54%)^**^
ICS	23 (11%)	24 (34%)^***^
ICS (monotherapy)	3 (1%)	5 (5%)
ICS + LABA	20 (10%)	31 (30%)
ICS + LABA + LAMA	0 (0%)	0 (0%)
Long-acting bronchodilator		
LABA	1 (0.5%)	5 (5%)^*^
LAMA	2 (1%)	17 (17%)^****^
Short-acting bronchodilator	15 (7%)	27 (26%)^***^

Data are expressed as mean ± SD for continuous variables and as *n* (%) for categorical variables: ^*^p < 0.05; ^**^p < 0.01; ^***^p < 0.001; ^****^p < 0.0001. *ACOS* Asthma-COPD overlap syndrome, *BMI* body mass index, *COP* cryptogenic organizing pneumonia, *DMARD* disease-modifying anti-rheumatic drugs, *FS* former smoker, *ILD* interstitial lung disease, *NS* non-smoker, *NSIP* non-specific interstitial pneumonia, *PF-ILD* progressive fibrosing interstitial pneumonia, *S* smoker, *UIP* usual interstitial pneumonia, *OCS* Oral corticosteroids, *ICS* inhaled corticosteroids, *LABA* long-acting B2 agonist, *LAMA* long-acting muscarinic antagonists. ^a^For the onset of RA and duration of disease, data are available for 188 non-ORA and 93 RA; ^b^For the ILD parameters, data were only reported if ILD was present; ^c^For asthma, data were available for 207 non-O-RA and 101 O-RA; ^d^For COPD, data were available for 207 non-O-RA and 98 O-RA; ^e^For pulmonary neoplasia at T0, data were available for 203 non-O-RA and 99 O-RA; ^f^For erosive RA, data were available for 101 non-O-RA and 43 O-RA.

### Pulmonary functional tests

Pulmonary functional tests are shown in Table 2. Spirometric values were significantly different between O-RA and non-O-RA patients. O-RA patients exhibited lower FEV1 (65% vs. 91% pred, *p* < 0.0001), FVC (86% vs. 93% pred, *p* < 0.01), which can be explained by hyperinflation linked to the obstruction, as well as DLCO (54% vs. 66% pred, *p* < 0.0001) but not KCO. By definition, all patients in the O-RA group were presenting a reduced Tiffeneau index (FEV1/FVC) under 70%, as commonly seen in COPD.

**Table 2:** Pulmonary function tests.

	RA	O-RA
	*n* = 207	*n* = 102
FEV-1 (L)	2.29 ± 0.72	1,63 ± 0,57^****^
FEV-1 (% pred.)	91 ± 20.1	65 ± 18.4^****^
FVC (L)	2.91 ± 0.91	2.70 ± 0.85
FVC (% pred.)	93 ± 21	86 ± 20^**^
FEV1/FVC (Tiffeneau index) (%)	79 ± 5.11	60 ± 8.38^****^
MEF25-75 (L/s)^1^	2.12 (1.54; 2.76)	0.78 (0.56; 1.16)^****^
MEF25-75 (%)^1^	76 (59; 94)	29 (22; 39)^****^
TLC (L)	4.86 ± 1.2	5.57 ± 1.35^****^
TLC (% pred.)	90 ± 17.8	100 ± 20^****^
FRC (L)^2^	3.02 ± 0.84	3.81 ± 1.19^****^
FRC (% pred.)^2^	103 ± 26	124 ± 33^****^
RV (L)	1.97 ± 0.65	2.79 ± 0.97^****^
RV (% pred.)	97 ± 29	128 ± 44^****^
DLCO (mmol.min^−1^.Kpa^−1^)	5.38 ± 1.94	4.35 ± 1.79^****^
DLCO (% pred.)	66 ± 19.9	54 ± 18.4^****^
KCO (mmol.min^−1^.Kpa^−1^.L^−1^)	1.23 ± 0.29	1.12 ± 0.39^**^
KCO (% pred.)	83 ± 20.1	78 ± 27.3
sGaw (L.sec^−1^.kPa^−1^.L^−1^)^1,2^	1.13 (0.76; 1.53)	0.69 (0.52; 0.98)^****^
sGaw (% pred.)^1,3^	75 (52; 99)	49 (41; 82)^*^

Data are expressed as mean ± SD or median (IQR) for continuous variables and as *n* (%) for categorical variables: ^*^p < 0.05; ^**^p < 0.01; ^***^p < 0.001; ^****^p < 0.0001. *DLCO* diffusing lung capacity of CO, *FEV1* forced expired volume in 1 s, *FRC* functional residual capacity, *FVC* forced vital capacity, *ILD* interstitial lung disease, *KCO* DLCO/Alveola ventilation, *TLC* total lung capacity, *sGaw* specific airway conductance, *MEF* maximum expiratory flow. ^1^Student test based on log-transformed data was performed for all values. ^2^For non-O-RA *n* = 175, O-RA *n* = 78. ^3^For non-O-RA *n* = 70, O-RA *n* = 15.

Inspiratory flow limitation related to dysfunction of the cricothyroid muscle of the larynx, which may be involved in RA patients, was not found in the medical record review (no dysphonia or inspiratory symptoms).

As usually identified in other obstructive airway diseases, O-RA patients had a significant reduction in their specific airway conductance as referred by the sGaw value compared to non-O-RA patients (0.69 and 1.13 l.sec^−1^.kPa^−1^.L^−1^, respectively, *p* < 0.0001), and small airway involvement assessed by maximum expiratory flow (MEF) 25/75 (29% vs. 76% pred, respectively, *p* < 0.0001). Supplementary Table S1 shows PFTs results according to each patient’s combination of pulmonary pathologies.

### Blood analysis

In the O-RA subgroup, we identified a significant decrease in lymphocyte count (*p* < 0.05) whereas CRP, fibrinogen, and neutrophils count were significantly increased (*p* < 0.001, *p* < 0.01, *p* < 0.01, respectively). We did not identify significant differences concerning total IgE and total eosinophil count (Table 3). Missing values are listed in Supplementary Table S2.

**Table 3:** Biological characteristics.

	Non-O-RA	O-RA
	*n* = 207	*n* = 102
Platelet count (10^3^/mm^3^)^1^	259 (217; 323)	274 (211; 324)
Hemoglobin (g/dL)	13.2 ± 1.51	13.1 ± 2.10
White blood cell count (10^3^/mm^3^)^1^	7.92 (6.02; 10.01)	8.67 (7.03; 10.2)
Absolute lymphocyte count (10^3^/mm^3^)^1^	1.91 (1.35; 2.41)	1.77 (1.18; 2.28)^*^
Lymphocyte (%)	25.9 ± 10.39	21.2 ± 9.37^***^
Absolute neutrophil count (10^3^/mm^3^)^1^	4.69 (3.31; 6.72)	5.91 (4.14; 7.56)^**^
Neutrophil (%)	62 ± 12.5	67 ± 11.7^***^
Absolute monocyte count (10^3^/mm^3^)^1^	0.56 (0.45; 0.75)	0.61 (0.47; 0.85)
Monocyte (%)	7.90 ± 3.42	7.79 ± 3.19
Absolute eosinophil count (10^3^/mm^3^)^1^	0.16 (0.10; 0.25)	0.15 (0.06; 0.25)
Eosinophil (%)^1^	2.00 (1.20; 3.50)	1.80 (0.80; 3.50)
Absolute basophil count (10^3^/mm^3^)^1^	0.04 (0.02; 0.05)	0.04 (0.02; 0.06)
Basophil (%)^1^	0.40 (0.30; 0.70)	0.50 (0.30; 0.70)
CRP (mg/L)^1^	4.64 (1.34; 12.6)	9.14 (2.73; 32.5)^***^
Fibrinogen (g/L)^1,2^	3.94 (3.20; 5.53)	4.75 (3.73; 5.62)^**^
Total IgE (U/L)^1,3^	34.2 (12.7; 97)	32.6 (18.2; 125)
ACPA (U/mL)^1,4^	41 (1; 200)	54 (7; 200)
RF (U/mL)^5^	19 (10–154)	34 (10–315)
	52.6% positive	61.0% positive
CCPA (U/mL)^5^	6.4 (0–135.21)	19 (10–154)

Data are expressed as mean ± SD or median (IQR) for continuous variables and as *n* (%) for categorical variables. ^*^p < 0.05; ^**^p < 0.01; ^***^p < 0.001; ^****^p < 0.0001. ^1^Student test based on log-transformed data was performed for all values. *ACPA* anti-citrullinated peptide antibody, *CCPA* cyclic citrullinated peptide antibodies, *RF* rheumatoid factor, *CRP* C-reactive protein. ^2^For non-O-RA *n* = 172, O-RA *n* = 93. ^3^For non-O-RA *n* = 54, O-RA *n* = 22. ^4^For non O-RA *n* = 73, O-RA *n* = 31. ^4,5^For non O-RA *n* = 167, O-RA *n* = 77. Rheumatoid factor was measured for non-O-RA and O-RA, respectively, at −0.6 ± 4.8 years and −1.6 ± 4.2 years from the first PFT.

### Survival analysis

The objective of this study was to predict the survival time for non-O-RA and O-RA patients. Therefore, time-dependent tools were required to evaluate these models. The predictive values of models have been evaluated using concordance statistics (Harrell’s C-statistics). The predictive accuracy of survival by O-RA is summarized in Supplementary Table S3 for both univariate and multivariate models.

An alternative is to compute the time-dependent ROC(*t*) curve and the associated AUC(*t*) for given survival thresholds *t* and to summarize it over time using the integrated AUC (iAUC). The AUC over time is represented in Supplementary Figure. Based on C-statistics or on iAUC, both multivariate models showed equivalent accuracy and they improved the accuracy from the univariate model.

In terms of survival analysis, we found that patients with O-RA had a significantly higher risk of death than those without obstruction (HR = 2.50; 95%CI 1.52–4.10, *p* < 0.001). The median survival time since the first PFT was 11.75 years in the O-RA group and was higher than 16 years in the non-O-RA group.

**Figure 1:**
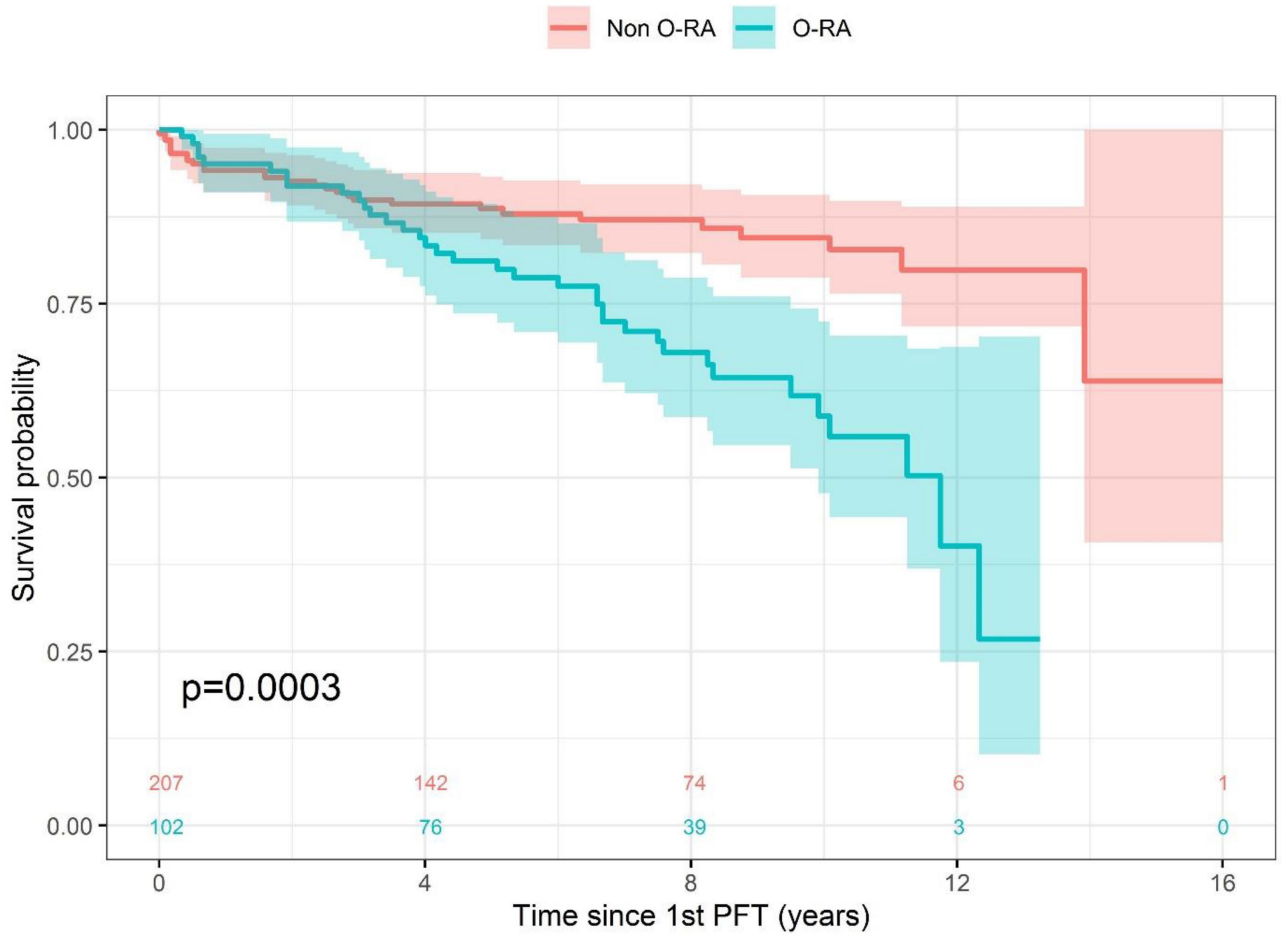
Survival analysis.

The survival probability at 8 years and 10 years was in the non-O-RA group at 87.1 and 84.5% and in the O-RA group at 68.0 and 58.8%, respectively (Figure 1).

### Multivariate survival analysis

In the univariate models, in addition to the obstructive character, all the parameters obtained from the fisrt PFT were significantly associated with an increased risk of death. This was also the case for age, ILD status, emphysema, COPD and active neoplasia at the beginning of the study.

The multivariate analysis performed on 301 patients, adjusting the model for the different comorbidities and demographic characteristics of the patients, confirmed that O-RA was an independent risk factor for mortality (HR 2.20; 95% CI 1.23–3.92, *p* < 0.01). ILD was also an independent risk factor for death (HR 1.86; 95% CI 1.06–3.27, *p* < 0.05), and age (HHR 1.04, 95% CI 1.01–10.7, *p* < 0.01). Active neoplasia was also an independent risk factor of increased mortality (HR 4.74, 95% CI 1.61–14.0, *p* < 0.01). Of note, we did not find any significantly increased mortality risk associated with bronchiectasis, or asthma (Table 4).

**Table 4:** Multivariate cox regression analysis.

		Univariate				Multivariate			
						*N* = 301			
Variable (1st PFT)	*N*	HR	95% CI		*p*-Value	HR	95% CI		*p*-Value
O-RA (Yes vs. No)	309	2.50	1.52	4.10	0.0003	2.20	1.21	4.00	0.0095
Age (years)	309	1.05	1.02	1.07	0.0002	1.04	1.01	1.07	0.007
Gender (M vs. F)	309	1.15	0.68	1.92	0.60	0.66	0.37	1.16	0.15
ILD (Yes vs. No)	309	1.69	1.01	2.82	0.047	1.86	1.06	3.27	0.031
Bronchiectasis (Yes vs. No)	309	1.49	0.89	2.49	0.13	1.44	0.84	2.48	0.19
Emphysema (Yes vs. No)	309	2.31	1.23	4.34	0.0091	1.90	0.94	3.82	0.073
Asthma (Yes vs. No)	308	0.96	0.44	2.11	0.92	0.77	0.32	1.82	0.55
Pulmonary neoplasia at T0 (Yes vs. No)	302	5.47	1.96	15.2	0.0012	4.74	1.61	14.00	0.0048
FEV-1 (L)	309	0.39	0.26	0.58	<0.0001				
FEV-1 (% pred.)	309	0.98	0.97	0.99	0.0002				
FVC (L)	309	0.50	0.37	0.69	<0.0001				
FVC (% pred.)	309	0.98	0.97	0.99	0.0003				
DLCO (mmol.min^−1^.Kpa^−1^)	280	0.71	0.59	0.84	<0.0001				
DLCO (% pred.)	280	0.97	0.95	0.98	<0.0001				
COPD	305	1.85	1.11	3.10	0.019				
GOLD score (only for O-RA)	100	1.20	0.80	1.81	0.38				
Smoking	129				0.83				
Former vs. no		0.92	0.42	1.99					
Yes vs. no		1.20	0.45	3.18					

Cox regression models were used to analyze the overall survival concerning groups and other parameters (FVC, DLCO, FEV-1, Age, Gender, RA-ILD, bronchiectasis, emphysema, asthma, neoplasia, and COPD). Results were considered significant at the 5% uncertainty level (*p* < 0.05).

## Discussion

The current study provides a comprehensive analysis of a cohort of patients suffering from RA retrospectively analyzed to better qualify the specific subgroup of those suffering from obstructive lung disease (O-RA). We found that obstruction is an independent risk factor for increased mortality. Knowing that lung disease is recognized as one of the major comorbidities in RA, and even if smoking abuse is widely known to be associated with an increased risk of RA, we cannot exclude that COPD can act as an isolated factor contributing to RA-associated morbimortality (8, 15, 16) through neutrophilic inflammation associated with this condition. Whereas more than 70% of our O-RA population had a significant smoking history, RA can probably also independently lead to obstruction through autonomous bronchiolar inflammation.

In our study, approximately two-thirds of the O-RA population could be described as suffering from COPD. We identified that in a cohort of 309 RA patients that benefited from PFT during their clinical follow-up, 33% of them were suffering from OLD. Those O-RA patients were displaying a higher TLC with hyperinflation based on RV evaluation associated with per definition a reduced FEV1/FVC ratio (17). DLCO was also significantly lower in the O-RA cohort compared to the other patients. The O-RA patients were also presenting a reduced specific conductance (sGaw) with increased FRC. Focusing on the mortality, O-RA patients presenting an overall increased mortality with a median survival of 11.8 years (HR 2.50, 95% CI 1.52–4.10) confirmed by the multivariate analysis (HR 2.20; 95% CI 1.23–3.92).

Several studies have identified that COPD was an independent risk factor for developing RA. This observation suggests that mucosal airway inflammation may increase the risk of RA (18). Of interest, a case-control study previously showed that both RA and COPD were exhibiting similar morbidities (18, 19). In a meta-analysis evaluating previous studies, it has been described that RA patients have a significantly increased risk of suffering from COPD with a pooled RR of 1.82 (95% CI = 1.55 to 2.10, *p* < 0.001) (10). This meta-analysis showed a prevalence of COPD in RA of 6.2% (95% CI = 4.1 to 8.3%). Of note, few have compared the mortality of these patients longitudinally. Our prevalence is probably higher than in other studies due to the retrospective aspect of our study, considering that PFT has probably been performed in symptomatic patients only.

We have recently shown in a 1,500 RA patients cohort that patients with ILD had a higher mortality risk compared to non-ILD RA patients (9). This study confirms that ILD is an independent predictor of mortality also for patients with O-RA.

The association of RA with COPD may have affected the severity of the disease. Indeed, COPD causes high mortality and may play a decisive role in all patients with various chronic diseases, including RA. Currently, we are prospectively evaluating whether COPD may be an interfering factor in the control of RA in a systematic manner.

Interestingly, we identified that patients with O-RA were exhibiting increased levels of CRP. As it was previously described in other studies, increased CRP and fibrinogen levels are known to be associated with increased overall mortality in COPD (20, 21) as well as with the risk of cardiovascular events (22). There is evidence that the inflammatory process in COPD is relevant to the development of cardiovascular disease and lung cancer (23). Therefore, we cannot avoid that common physiopathological processes may be shared by both COPD and O-RA patients.

Whereas COPD is known to be the major cause of OLD, we cannot certify that in our cohort none of them was suffering from obliterans bronchiolitis (also previously referred to as constrictive bronchiolitis), which is clinically known to be more severe and often quickly fatal in RA.

The retrospective aspect of this study must be disclosed as a significant bias. Indeed, patients who have undergone PFT are thought to be more symptomatic than others or are suspected of suffering from an OLD. Therefore, the prevalence of O-RA might have been overestimated. We cannot avoid that some of them can also experience bronchiolitis associated with RA (15). This can overestimate the proportion of O-RA patients in a RA cohort. This bias was mitigated in our study as the non-O-RA cohort was similarly recruited.

Surprisingly, in our O-RA cohort, we found that only 41.4% of the population was treated with bronchodilators, which are the cornerstone of COPD treatment. Thus, COPD patients in this RA cohort were under-treated. However, the majority of O-RA patients aerosol-treated would have benefited in our study from those therapies based on the GOLD recommendation. COPD and RA have similar complications over time. Indeed, it appears that these two situations lead to progressive musculoskeletal degradation linked to a significant deterioration in the quality of life and a functional decline. In this context, both drug management and musculoskeletal rehabilitation remain imperative to minimize long-term complications.

One other limitation of this study out of its retrospective aspect is the lack of information concerning the occurrence of incidental respiratory infection favored by immunosuppressive therapies. COPD patients are at higher risk to develop lower respiratory tract infections. In this specific population, adding bronchodilator therapy is a key factor to reduce acute exacerbations. The multivariate analysis did not include immunosuppressive status since patients had benefited from multiple specific therapies that can modify the interpretability of those results. Moreover, we did not have information on the cardiovascular status of the patients, which is known to be also associated with an increased risk of mortality. We were not able to retrieve from the medical records frequent comorbidities such as diabetes, renal or hepatic insufficiency, and arterial hypertension, because this study was retrospective, and we did not have a reliable and dedicated evaluation of these parameters.

Many patients were referred to our center, which is a tertiary clinical center where the patients did not benefit from other examinations than rheumatologic ones, during which these points were not systematically identified as present or absent.

In conclusion, patients with combined obstructive lung disease and rheumatoid arthritis exhibit a characteristic functional profile with reduced FEV-1, small airway involvement, reduced sGaw, hyperinflation, and lower carbon monoxide diffusing capacity of the lung. Our study identified that patients with O-RA were at higher risk of death compared to other RA patients. OLD in RA seems to be an independent risk factor of mortality based on our data potentially associated with an underlying inflammatory process similar to what is seen in COPD.

Therefore, patients with RA have to be considered for specific and dedicated pulmonary evaluation to treat both ILD and OLD. Spirometry is a key indicator of RA-associated lung disease that can modify a patient’s outcome. Further prospective longitudinal studies will have to determine its implication in patients’ management and follow-up and particularly the need for an early diagnosis to propose appropriate bronchodilator therapy.

## Data availability statement

The datasets generated and/or analyzed during the current study are not publicly available because these data are considered sensitive but are available from the corresponding author upon reasonable request.

## Ethics statement

The studies involving human participants were reviewed and approved by Ethics Committee of the University Hospital of Liège (Belgian Number: B707201422832; ref.: 2022/52). Written informed consent for participation was not required for this study in accordance with the national legislation and the institutional requirements.

## Author contributions

JG, MM, and RL conceived the study. JG, ME, and LS developed the study methodology. MH and MT collected data and validated the data. AD, A-NF, FG, PC, CR, OM, LG, and OG acquired data. JG, M-SN, PM, and J-LC analyzed the data. MH, ME, and LS made the statistical analysis. JG, MH, ME, LS, and MW wrote the manuscript. MM and RL coordinated the research activity. All authors contributed to the article and approved the submitted version.

## Conflict of interest

JG reports personal fees for the advisory board, work, and lectures from Boehringer Ingelheim, Janssens, GSK, Roche, AstraZeneca, and Chiesi, non-financial support for meeting attendance from AstraZeneca, Chiesi, Roche, Boehringer Ingelheim, and Janssens. He is in the permanent SAB of Radiomics (Oncoradiomics SA) for the SALMON trial without any specific consultancy fee for this work. He is a co-inventor of one issued patent on radiomics licensed to Radiomics (Oncoradiomics SA).

The remaining authors declare that the research was conducted in the absence of any commercial or financial relationships that could be construed as a potential conflict of interest.

## Publisher’s note

All claims expressed in this article are solely those of the authors and do not necessarily represent those of their affiliated organizations, or those of the publisher, the editors and the reviewers. Any product that may be evaluated in this article, or claim that may be made by its manufacturer, is not guaranteed or endorsed by the publisher.

## Figures and Tables

**Table:** 

ACOS	Asthma-COPD overlap syndrome
ACPA	anti-citrullinated peptide antibody
BMI	body mass index
CCPA	cyclic citrullinated peptide antibodies
COP	cryptogenic organizing pneumonia
COPD	chronic obstructive lung disease
DLCO	diffusing lung capacity of CO
DMARD	disease-modifying anti-rheumatic drugs
FEV1	forced expired volume in 1 s
FRC	functional residual capacity
FS	former smoker
FVC	forced vital capacity
HRCT	high-resolution computed tomography
ICS	inhaled corticosteroids
ILD	interstitial lung disease
LABA	long-acting B2 agonist
LAMA	long-acting muscarinic antagonists
MEF	maximum expiratory flow
NS	non-smoker
NSIP	non-specific interstitial pneumonia
OCS	Oral corticosteroids
OLD	obstructive lung disease
O-RA	obstructive-rheumatoid arthritis
PF-ILD	progressive fibrosing interstitial pneumonia
PFT	pulmonary functional test
RA	rheumatoid arthritis
RF	rheumatoid factor
S	smoker
sGaw	specific conductance
TLC	total lung capacity
UIP	usual interstitial pneumonia

## Supplementary material

SUPPLEMENTARY FIGURE S1 Comparison of time-dependent AUC(t) for univariate or multivariate models for survivval analysis.
