# Supplementary figures and images for: Airflow obstruction as a marker of adverse prognosis in rheumatoid arthritis
Source: Front Med (Lausanne). 2023 Mar 9;10:1063012. doi: 10.3389/fmed.2023.1063012 (PMC10033600; doi:10.3389/fmed.2023.1063012)

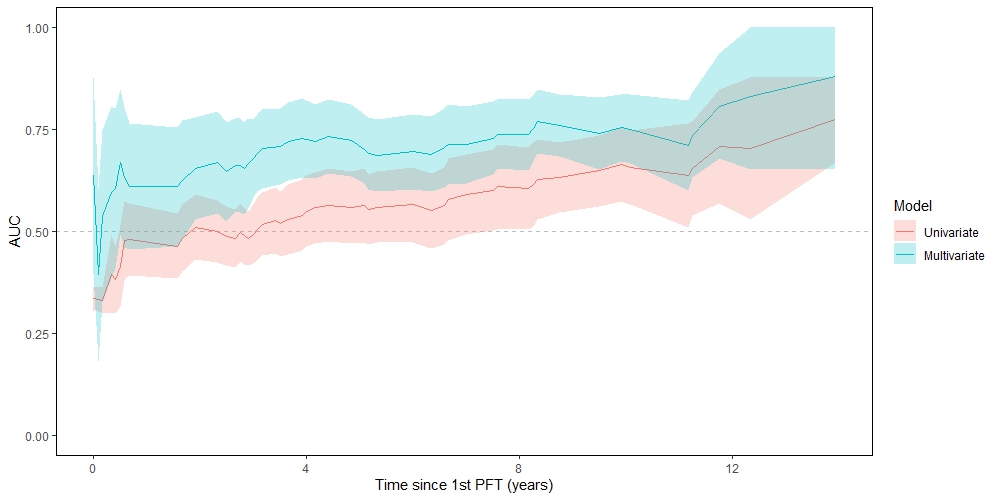

Supplement: SUPPLEMENTARY FIGURE S1 — Comparison of time-dependent AUC(t) for univariate or multivariate models for survivval analysis. [file Image_1.JPEG]
