# Supplementary material for: Airflow obstruction as a marker of adverse prognosis in rheumatoid arthritis
Source: Front Med (Lausanne). 2023 Mar 9;10:1063012. doi: 10.3389/fmed.2023.1063012 (PMC10033600; doi:10.3389/fmed.2023.1063012)
Supplement: Supplementary file 2 [file Table_1.docx]

S1. Sub-classification of pulmonary functional tests according to lung pathologies

|  | **Non-O-RA** | **Non O-RA + ILD + Bronchiectasis** | **Non-O-RA + ILD** | **nonO-RA + Bronchiexcatsis** | **O-RA** | **O-RA + Bronchiectasis** | **O-RA + ILD** | **O-RA + ILD + Bronchiectasis** |
| --- | --- | --- | --- | --- | --- | --- | --- | --- |
| **n** | 118 | 26 | 33 | 30 | 69 | 15 | 12 | 6 |
| **FEV-1 (L)** | 2.41 ± 0.73 | 2.17 ± 0.66 | 2.3 ± 0.67 | 1.92 ± 0.7 | 1.68 ± 0.56 | 1.36 ± 0.68 | 1.6 ± 0.44 | 1.78 ± 0.69 |
| **FEV-1 (% pred)** | 93 ± 19 | 85 ± 17 | 91 ± 18 | 85 ± 28 | 66 ± 17 | 57 ± 26 | 70 ± 12 | 68 ± 21 |
| **FVC** | 3.05 ± 0.89 | 2.75 ± 0.89 | 2.9 ± 0.87 | 2.48 ± 0.93 | 2.76 ± 0.83 | 2.36 ± 0.96 | 2.6 ± 0.74 | 3.12 ± 0.84 |
| **FVC (% pred)** | 97 ± 19 | 85 ± 17 | 92 ± 19 | 89 ± 29 | 87 ± 19 | 78 ± 26 | 91 ± 20 | 93 ± 18 |
| **Tiffeneau index (%)** | 79 ± 5 | 80 ± 7 | 80 ± 5 | 78 ± 5 | 61 ± 8 | 57 ± 11 | 62 ± 7 | 56 ± 13 |
| **DEM25_75 (L)** | 2.31 ± 0.91 | 2.13 ± 0.84 | 2.4 ± 0.81 | 1.73 ± 0.68 | 0.9 ± 0.4 | 0.7 ± 0.45 | 0.86 ± 0.32 | 0.88 ± 0.42 |
| **DEM25_75_(% pred)** | 79 ± 27 | 83 ± 33 | 90 ± 37 | 68 ± 25 | 32 ± 13 | 25 ± 15 | 32 ± 11 | 34 ± 18 |
| **TLC (L)** | 466 ± 158 | 386 ± 184 | 529 ± 462 | 453 ± 144 | 508 ± 199 | 542 ± 264 | 454 ± 172 | 649 ± 324 |
| **TLC (% pred)** | 94 ± 15 | 78 ± 17 | 97 ± 58 | 88 ± 20 | 100 ± 19 | 108 ± 19 | 93 ± 20 | 107 ± 41 |
| **FRC (L)** | 282 ± 110 | 253 ± 109 | 352 ± 456 | 257 ± 130 | 356 ± 123 | 386 ± 154 | 320 ± 131 | 387 ± 270 |
| **FRC_(% pred)** | 106 ± 24 | 89 ± 25 | 124 ± 114 | 98 ± 22 | 123 ± 28 | 144 ± 32 | 105 ± 33 | 128 ± 56 |
| **RV (L)** | 188 ± 77 | 165 ± 87 | 241 ± 447 | 196 ± 79 | 258 ± 121 | 340 ± 137 | 224 ± 127 | 198 ± 236 |
| **RV (%pred)** | 102 ± 29 | 79 ± 33 | 117 ± 159 | 98 ± 24 | 131 ± 48 | 163 ± 36 | 110 ± 40 | 128 ± 82 |
| **DLCO (mmol.min^-1^.Kpa^-1^)** | 5.64 ± 1.88 | 4.95 ± 1.85 | 4.77 ± 1.64 | 5.42 ± 2.37 | 4.38 ± 1.82 | 4.47 ± 2.33 | 4.01 ± 1.37 | 4.64 ± 1.51 |
| **DLCO(% pred)** | 69 ± 19 | 61 ± 20 | 60 ± 19 | 67 ± 21 | 54 ± 18 | 56 ± 24 | 53 ± 15 | 59 ± 20 |
| **sGaw (L.sec^-1^.kPa^-1^.L^-1^)** | 105 ± 58 | 120 ± 71 | 124 ± 58 | 91 ± 51 | 77 ± 46 | 51 ± 33 | 81 ± 40 | 80 ± 41 |
| **sGaw_(% pred)** | 76 ± 37 | 104 ± 58 | 90 ± 35 | 60 ± 33 | 60 ± 36 | 0 ± 0 | 86 ± 0 | 55 ± 8 |

S1. Data are expressed as mean ± SD or median (IQR) for continuous variables and as n (%) for categorical variables. *DLCO* diffusing lung capacity of CO, *FEV1* forced expired volume in 1s, *FRC* functional residual capacity, *FVC* forced vital capacity, *ILD* interstitial lung disease, *KCO* DLCO/Alveola ventilation, *TLC* total lung capacity, *sGaw* specific airway conductance, *MEF* maximum expiratory flow.
