# Supplementary material for: Airflow obstruction as a marker of adverse prognosis in rheumatoid arthritis
Source: Front Med (Lausanne). 2023 Mar 9;10:1063012. doi: 10.3389/fmed.2023.1063012 (PMC10033600; doi:10.3389/fmed.2023.1063012)
Supplement: Supplementary file 3 [file Table_2.docx]

S2 Evaluation of the predictive accuracy of the survival model of O-RA using Harrell’s C-statistics with Standard Error (SE) and integrated AUC (iAUC).

|  |  | **O-RA (yes vs no)** | | | |  |  |  |
| --- | --- | --- | --- | --- | --- | --- | --- | --- |
| **Model** | **N** | **HR** | **95%** | **CI** | **p-value** | **C (%)** | **SE (%)** | **iAUC (%)** |
| Univariate | 309 | 2.50 | 1.52 | 4.10 | 0.0003 | 57.6 | 3.31 | 63.0 |
| Multivariate model  (7 cofactors)^1^ | 301 | 2.20 | 1.21 | 4.00 | 0.0095 | 69.2 | 3.64 | 75.9 |

C: concordance factor obtained with Harrell’s C-statistics. Cofactors used in the multivariate model were Age, Gender, ILD, Bronchiectasis, Emphysema, Asthma, and Neoplasia at T0.
