# Supplementary material for: Airflow obstruction as a marker of adverse prognosis in rheumatoid arthritis
Source: Front Med (Lausanne). 2023 Mar 9;10:1063012. doi: 10.3389/fmed.2023.1063012 (PMC10033600; doi:10.3389/fmed.2023.1063012)
Supplement: Supplementary file 4 [file Table_3.docx]

S3. Missing values listing

|  | Non-O-RA | O-RA | Non-O-RA | O-RA |
| --- | --- | --- | --- | --- |
|  | Missing value : n | | Missing value: % | |
| Table 1. Patient characteristics |  |  |  |  |
| Smokers NS/FS/CS (%) | 98 | 44 | 47% | 43% |
| Duration of RA, years | 19 | 9 | 9% | 9% |
| Erosive RA | 101 | 43 | 49% | 42% |
| Treatments | 44 | 11 | 21% | 11% |
| Table 2. Pulmonary function tests |  |  |  |  |
| TLC (L) | 9 | 6 | 4% | 6% |
| TLC (% pred.) | 9 | 6 | 4% | 6% |
| FRC (L) | 9 | 6 | 4% | 6% |
| FRC (% pred.) | 9 | 6 | 4% | 6% |
| RV (L) | 9 | 6 | 4% | 6% |
| RV (% pred.) | 9 | 6 | 4% | 6% |
| DLCO (mmol.min-1.Kpa-1) | 16 | 13 | 8% | 13% |
| DLCO (% pred.) | 16 | 13 | 8% | 13% |
| KCO (mmol.min-1.Kpa-1.L-1) | 16 | 13 | 8% | 13% |
| KCO (% pred.) | 16 | 13 | 8% | 13% |
| sGaw (L.sec-1.kPa-1.L-1) | 32 | 24 | 15% | 24% |
| sGaw (% pred.) | 137 | 87 | 66% | 85% |
| Table 3. Biological characteristics |  |  |  |  |
| Platelet count (10³/mm³) | 2 | 0 | 1% | 0% |
| Hemoglobin (g/dL) | 2 | 0 | 1% | 0% |
| White Blood Cell count (10³/mm³) | 2 | 0 | 1% | 0% |
| Absolute Lymphocyte count (10³/mm³) | 2 | 0 | 1% | 0% |
| Lymphocyte (%) | 2 | 0 | 1% | 0% |
| Absolute Neutrophil count (10³/mm³) | 2 | 0 | 1% | 0% |
| Neutrophil (%) | 2 | 0 | 1% | 0% |
| Absolute Monocyte count (10³/mm³) | 2 | 0 | 1% | 0% |
| Monocyte (%) | 2 | 0 | 1% | 0% |
| Absolute Eosinophil count (10³/mm³) | 2 | 0 | 1% | 0% |
| Eosinophil (%) | 2 | 0 | 1% | 0% |
| Absolute Basophil count (10³/mm³) | 2 | 0 | 1% | 0% |
| Basophil (%) | 2 | 0 | 1% | 0% |
| CRP (mg/L) | 2 | 0 | 1% | 0% |
| Fibrinogen (g/L) | 35 | 9 | 17% | 9% |
| Total IgE (U/L) | 153 | 80 | 74% | 78% |
| ACPA (U/mL) | 134 | 71 | 65% | 70% |
| RF (U/mL) | 40 | 25 | 19% | 25% |
| CCPA (U/mL) | 40 | 25 | 19% | 25% |
